# Explained Deep Learning Framework for COVID-19 Detection in Volumetric CT Images Aligned with the British Society of Thoracic Imaging Reporting Guidance: A Pilot Study

**DOI:** 10.1007/s10278-025-01444-3

**Published:** 2025-02-26

**Authors:** Shereen Fouad, Muhammad Usman, Ra’eesa Kabir, Arvind Rajasekaran, John Morlese, Pankaj Nagori, Bahadar Bhatia

**Affiliations:** 1 School of Computer Science and Digital Technologies, Aston University, Birmingham, UK; 2 Sandwell and West Birmingham Hospitals NHS Trust, West Birmingham, UK

**Keywords:** COVID-19, British Society of Thoracic Imaging, Deep learning, Multi-class classification, Medical image analysis, Explainable AI

## Abstract

In March 2020, the British Society of Thoracic Imaging (BSTI) introduced a reporting guidance for COVID-19 detection to streamline standardised reporting and enhance agreement between radiologists. However, most current DL methods do not conform to this guidance. This study introduces a multi-class deep learning (DL) model to identify BSTI COVID-19 categories within CT volumes, classified as ‘Classic’, ‘Probable’, ‘Indeterminate’, or ‘Non-COVID’. A total of 56 CT pseudoanonymised images were collected from patients with suspected COVID-19 and annotated by an experienced chest subspecialty radiologist following the BSTI guidance. We evaluated the performance of multiple DL-based models, including three-dimensional (3D) ResNet architectures, pre-trained on the Kinetics-700 video dataset. For better interpretability of the results, our approach incorporates a post-hoc visual explainability feature to highlight the areas of the image most indicative of the COVID-19 category. Our four-class classification DL framework achieves an overall accuracy of 75%. However, the model struggled to detect the ‘Indeterminate’ COVID-19 group, whose removal significantly improved the model’s accuracy to 90%. The proposed explainable multi-classification DL model yields accurate detection of ‘Classic’, ‘Probable’, and ‘Non-COVID’ categories with poor detection ability for ‘Indeterminate’ COVID-19 cases. These findings are consistent with clinical studies that aimed at validating the BSTI reporting manually amongst consultant radiologists.

## Introduction

COVID-19 is a respiratory illness caused by the SARS-CoV-2 virus. It is characterised by a range of symptoms from mild respiratory distress to severe pneumonia, and it has resulted in a global pandemic originating in December 2019 with significant public health implications. Due to mutations in COVID-19, multiple variants are continuously evolving, putting the public’s health at risk of increased transmissibility and ongoing health complications. At the time of writing, a new variant BA.2.86 has emerged with concerns of increased cases [27]. The Reverse Transcriptase-Polymerase Chain Reaction (RT-PCR) testing kit has been used as a rapid diagnostic tool to identify active COVID-19 infections. However, it suffers from several drawbacks, including limited sensitivity, inconsistent efficacy, and the likelihood of false negatives in COVID-19-positive patients.

**Fig. 1:**
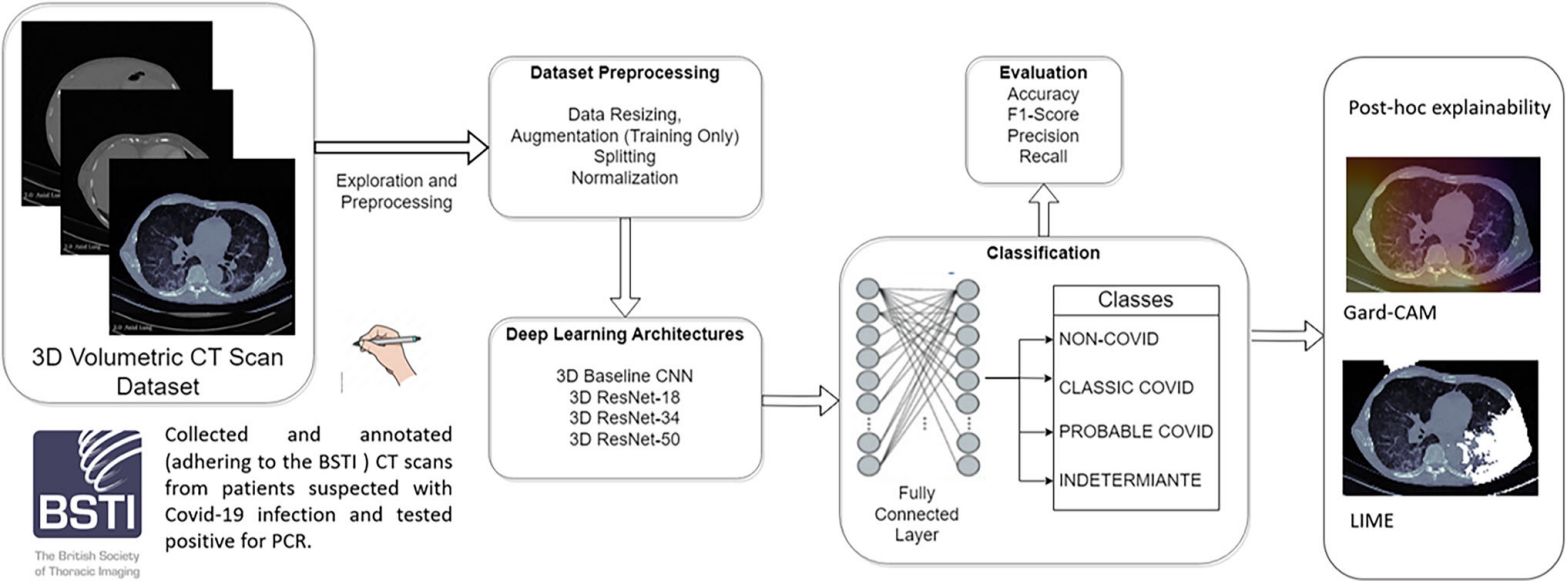
Block-diagram with an overview of the proposed method

Identifying COVID-19 infection from CT images is a challenging task for radiologists due to the variability in manifestations, non-specific imaging features, and confounding respiratory conditions. In March 2020, the British Society of Thoracic Imaging (BSTI) [8, 12] introduced a reporting guidance to streamline standardised reporting and enhance agreement between radiologists **in the UK**. This technique categorises chest CT images into four COVID-19 groups based on radiological findings: ‘Classic’ (100% confidence for COVID-19), ‘Probable’ (71–99% confidence for COVID-19), ‘Indeterminate’ (<70% confidence for COVID-19) and ‘Non-COVID’-19 (70% confidence for alternative). Medical professionals and radiologists have found this reporting system a valuable tool in aiding clinical assessment [13, 23]. **This reporting system has been shown to be effective when used by reporting radiographers, with high accuracy in matching report descriptions to the correct codes (98.8% in** [33]**). **

In recent years, artificial intelligence (AI), particularly Deep learning (DL), has emerged as a promising tool for the automatic detection of COVID-19 using chest CT images. These tools have the capacity to learn from labelled training data in order to infer diagnostic decisions on unseen data input. The training data is often hand-labelled by experts (radiologists) to indicate the presence or absence of COVID-19 infection, classifying images into ‘COVID-19 Negative’ and ‘COVID-19 Positive’. Several DL approaches have been proposed in the literature for the detection of COVID-19 infections using volumetric CT images [17, 26, 30, 31, 34]. However, most of the current methods have been trained using datasets that do not adhere to the standard and widely adopted **(in the UK)** BSTI guidance for reporting radiologists. The predominant focus of current DL approaches remains on binary classification for COVID-19 cases (positive or negative) (e.g. [32, 36]). This limitation is mainly due to the shortage of appropriately annotated datasets for BSTI as well as the complexity of training three-dimensional (3D) DL models for multi-class classification problems. Automatic detection of BSTI categories in CT volumes is crucial as it highlights the degree of radiological certainty about the disease and supports the treatment pathway.

Another drawback of current 3D DL-based methods for COVID-19 detection is the lack of explainability for medical and radiology stakeholders. The black-box nature and high complexity of these methods have restricted their acceptance. Explainable AI (XAI) is an emerging concept that deals with the implementation of techniques that improve the transparency, interpretability, and trust of complex AI methods [3, 9, 10]. Current XAI tools for radiology imaging focus mostly on providing post-hoc visual interpretation of the DL results by highlighting the important areas (regions of interest) in the image that drive the DL decision using saliency (heat) maps [29]. In the context of COVID-19 detection using CT images, visual explainability is important to highlight COVID-19 infection markers, such as ground-glass opacities (GGOs) and consolidations. This feature allows clinicians and radiologists to see which patterns in the image are influencing the model’s decision, therefore improving trust in the DL findings.

This study aims to address the above gaps by proposing an explainable multi-class classification DL framework for detecting COVID-19 in volumetric CT scans using BSTI guidance. Figure 1 presents the different steps performed in this study. To the best of our knowledge, the automatic (DL-based) detection of BSTI COVID-19 groups has not been investigated in the literature. Our contributions can be summarised as follows:

- We gathered and annotated (adhering to the BSTI) a unique set of 56 CT scans from patients suspected with COVID-19 infection and tested positive for PCR.
- We proposed a multi-class classification DL framework for classifying CT volumes into four BSTI groups: ‘Classic’ COVID-19, ‘Probable’ COVID-19, ‘Indeterminate’, and ‘Non-COVID’-19. We studied the performance of four 3D DL-based models, whose hyperparameters have been appropriately optimised.
- We demonstrated the benefit of transfer learning in providing more accurate prediction when compared to a baseline convolutional neural networks (CNN) model.
- We investigated the impact of ‘Indeterminate COVID-19’ BSTI category on the performance of the DL detection framework.
- We applied a post-hoc visual explainable tool to the classification output to validate the prediction using Grad-CAM (Gradient-weighted Class Activation Mapping) [29] algorithm.

## Related Work

During the COVID-19 pandemic, a huge number of open-access imaging datasets has emerged to facilitate the development of automated tools for detecting COVID-19 infection. The datasets involve chest images captured through X-ray or CT modalities. However, most of the open-access datasets have been annotated using binary labels (COVID-19 or ‘Non-COVID’-19) [32, 36]. Other datasets have been annotated to multiple classes to enable the development of DL models for detecting various chest diseases or COVID-19 severity levels (e.g. [7, 15, 21, 22, 28, 30]). To our knowledge, none of the current open-access CT imaging datasets for COVID-19 detection strictly adhere to the reporting of the BSTI [8, 12]. The dataset that closely aligns with the BSTI is provided by the Society of Imaging Informatics in Medicine (SIIM) for a Kaggle competition [21]. However, it comprises X-ray images rather than CT imaging modality.

There has been a growing interest in utilising DL tools for the detection of COVID-19 through the analysis of CT and X-ray images. Most existing DL methods primarily focus on binary class detection, distinguishing positive from negative cases. For instance, Yang et al. [35] introduced a DL approach that detected COVID-19 using 295 High-Resolution CT (HRCT) images, employing a pre-trained DenseNet architecture. Kim et al. [20] introduced the ‘FCONet’ (FastTrack COVID-19 classification network) DL framework using CT images of both COVID-19 and healthy cases. They studied several pre-trained DL models such as VGG16, ResNet-50, Inception-v3, and Xception. However, it is worth noting that these studies were conducted on 2D CT slices, potentially limiting their ability to capture the full context of 3D images. As highlighted by [4], CT volumes demonstrate higher sensitivity and specificity in COVID-19 detection compared to 2D image modalities. This is due to their ability to get 3D insights into the nature and location of lung issues, particularly ground-glass opacities (GGOs), thereby enabling more precise diagnoses. **A recent work in** [2]**, proposed a novel DL framework, ResNet-50U-Net, to enhance GGO segmentation accuracy by leveraging a pretrained ResNet-50 for improved feature extraction. Tested on 62 annotated COVID-19 volumetric CT images, this model outperformed standard U-Net and DenseNet-121U-Net architectures, achieving a Dice similarity score of 0.71, Precision of 0.63, and Recall of 0.83.**

In the context of multi-class COVID-19 classification using chest CT scans, several studies have been presented that focused on detecting COVID-19 severity. For instance, Shiri et al. [30] categorised COVID-19 CT scans into four severity levels using 1110 patient images, applying two feature selection methods and a multinomial logistic regression classifier, reaching 92% accuracy. **A multi-stage architecture has been developed in** [28] **for COVID-19 classification, infection region identification, and severity assessment. It isolates lung regions, differentiates COVID-19 from pneumonia and normal images using a fuzzy rank-based ensemble of pre-trained models, and quantifies infection severity to categorise cases into four levels. Another recent study in** [15] **utilised machine learning and statistical atlas-based methods to investigate lung shape changes in COVID-19 patients and their association with disease severity. Using a large dataset (*N*** **= 3443), the study defined three populations—healthy, mild COVID-19, and severe COVID-19—and analysed baseline chest CT scans. Significant lung shape differences were identified along the mediastinal surfaces across all severity levels, with additional differences on basal surfaces between healthy and severe cases. A 3D residual convolutional network integrating lung shape changes and GGOs further demonstrated a strong association with COVID-19 severity. A study** [7] **utilising COVID-19 data from two Manipal hospitals achieved a maximum testing accuracy of 95% using classifiers with nature-inspired feature selection algorithms. The dataset included 599 non-severe COVID patients and 300 severe COVID patients. Explainable AI techniques identified six key biological markers as critical for prediction.**

Other studies have performed multi-class classification to differentiate between various chest diseases. For example, Singh et al. [31] introduced a DL ensemble framework for classifying patients into COVID-19, pneumonia, tuberculosis, and normal, using 11,494 scans. They utilised Densely Connected Convolutional Networks (DCCNs), ResNet152V2, and VGG16, achieving up to 98% accuracy. Wu et al. [34] presented Covid-AL, a hybrid structure for COVID-19 diagnosis from CT images using weakly supervised DL. Their dataset comprised 962 CT images divided into COVID-19, common pneumonia, and normal classes. COVID-AL utilised a 2D U-Net for lung area segmentation and a 3D residual network for COVID-19 detection, achieving an accuracy rate of 86.60%. [17] assessed four neural networks using a combination of public digital chest X-rays and CT scans to increase the dataset size. A comparative analysis was conducted using four DL architectures to classify images into Normal, COVID-19, Pneumonia, and Lung cancer. Results reveal that the VGG19-CNN model excelled with over 98% in accuracy, recall, and precision.

Few studies have performed multi-class classification to differentiate between various appearances of COVID-19 infections in the lung. Muhammad et al. [26] proposed a classification framework using the SIIM X-ray dataset, comprising 6,054 cases, for categorising images into negative, typical, Indeterminate, or atypical appearances of COVID-19 pneumonia. Three different approaches were studied including a baseline DenseNet model, multi-task learning, and self-supervised learning. However, the accuracy obtained was relatively lower (64%) when compared to earlier studies.

Despite these multi-class classification DL methods, no study has specifically focused on detecting BSTI guidance for classifying COVID-19 groups in CT volumes. The automated identification of BSTI categories within CT volumes is essential as it signifies the level of radiological certainty regarding the COVID-19 disease.

## Data Collection and Preparation

In this study, the dataset was collected in 2020 at the Sandwell and West Birmingham National Health System Trust (SWBNHST) hospitals in the UK. The study was reviewed and approved by the ethics committee at Aston University (No. EPS21006 and 234700-51). All procedures were conducted in compliance with relevant guidelines and regulations.

**The dataset consists of 56 pseudonymised chest non-contrast CT studies, captured using Siemens CT scanners (Flash, AS, and Drive), which are 1–2 mm slice thickness.** The CT scans were obtained when the clinical referrer suspected the presence of COVID-19 in the patients, owing to confirmatory positive tests for PCR. The appearance of viral pneumonia exhibits significant heterogeneity, and COVID-19 was no exception. CT in COVID-19 shows typical findings of ground-glass opacity (GGOs), peripheral consolidation, or a combination of both. To annotate the dataset, a fellowship-trained subspecialty chest radiologist manually annotated the slices, utilising ITK-SNAP v3.8.0. Radiologists can diagnose COVID-19 pneumonia by analyzing the presence of certain features and evaluating their impact on different lung regions. The dataset includes labeled GGO markers, such as patchy, diffuse, crazy paving, consolidation (band-like, demarked), honeycomb cysts, and reticular patterns. Their potential distribution is described in the Table 1 below:

**Table 1:** Disease distribution and extent of abnormality

Disease distribution	Extent of abnormality
Bilateral	$\le$ 25%
Unilateral	26–50%
Patchy	51–75%
Diffuse	> 75%

**Fig. 2:**
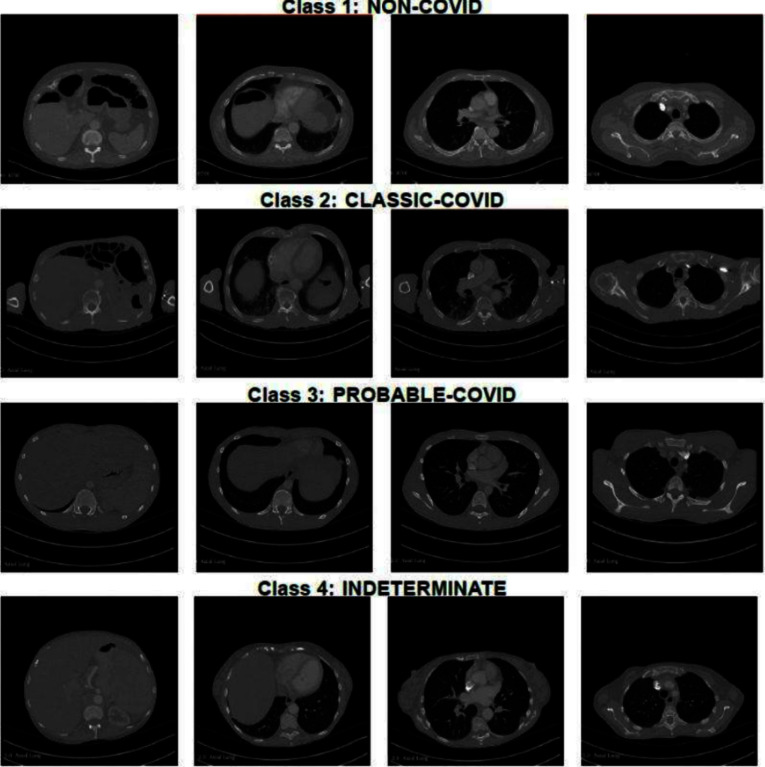
Example CT image slices for each of the four BSTI COVID-19 groups (highlighted at the top of each row)

The dataset was labeled for COVID-19 following the BSTI [8, 12], version 2 (March 2020). Table 2 offers an overview of the sample size and descriptions for each class within the dataset. Figure 2 displays example image slices extracted from 2D images for each class in the dataset. The studies were pseudonymised on-premise within the hospital using open-source tools, including the Radiological Society of North America Clinical Trials Processor (CTP).^1^

**Table 2:** Dataset Information, adapted from BSTI$^{2}$. [8, 12]

Class	Sample size	Confidence	Description
‘Classic’ COVID	10	100% confidence for COVID	**(i) ** Lower lobe predominant, peripheral predominant, multiple, bilateral foci of GGO,**(ii)** Crazy-paving, **(iii)** Peripheral consolidation, **(vi)** Air bronchograms, **(v)** Reverse halo/perilobular pattern
‘Probable’ COVID	21	71–99% confidence for COVID	**(i)** Lower lobe predominant mix of bronchocentric and peripheral consolidation, **(ii)** Reverse halo/perilobular pattern, **(iii)** GGO scarce
Indeterminate	10	< 70% confidence for COVID	**(i)** Does not fit into definite, probable or Non-COVID, **(ii)** Manifests above patterns, but the clinical context is wrong, or suggests an alternative diagnosis (e.g. an interstitial lung disease in a connective tissue disease setting)
‘Non-COVID’	15	70% confidence for alternative	**(i)** Lobar pneumonia, **(ii)** Cavitating infections, **(iii)** Tree-in bud/ centrilobular nodularity, **(vi)** Lymphadenopathy, effusions, **(v)** Established pulmonary fibrosis

Available from: https://www.bsti.org.uk/media/resources/files/BSTI_COVID-19_Radiology_Guidance_version_2_16.03.20.pdf

**Fig. 3:**
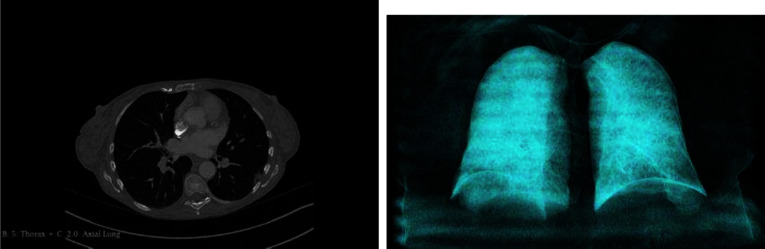
Axial slice and 3D rendered airway

The chest CT scans for each patient were exported in the Digital Imaging and Communications in Medicine (DICOM) format. All images are in grayscale with single-channel dimensions and a resolution of 512 $\times$ 512, including pixel data in Hounsfield Units (HU). The number of slices in each volumetric CT image varied, with a median of 314 slices per 3D CT scan. The scanning parameters, including pixel spacing and slice thickness, exhibited slight variations between each CT scan. Figure 3 provides an example of a single axial slice and an image rendering displaying the lung’s air passages. **To ensure privacy and anonymity, the data has undergone a process of anonymization using the Medical Imaging Resource Centre (MIRC) Clinical Trials Processor (CTP) method.**

### Data Preprocessing

The primary aim of the preprocessing phase is to generate high-quality images, ensuring their suitability for training and validation in the employed CNN-based architectures. CT scans are typically represented in Hounsfield Units (HU), which quantifies tissue radiodensity relative to that of water. Initially, we read 2D DICOM slices for each 3D volume and converted them from their raw pixel values to HU. Subsequently, a filtering process was applied to the 2D CT slices to eliminate non-lung structures, such as skin, bone, or scanner-related artifacts. This filtering operation served to improve the quality of our analysis and retain only the image components relevant to the lungs, which typically fall within the HU range of −1000 to 400. All CT slices, originally sized at 512 $\times$ 512 pixels for each CT Scan, were uniformly resised to 128 $\times$ 128 pixels on both the *x* and *y* dimensions. This resizing was undertaken to significantly reduce the memory requirements for accommodating the images within the GPU memory. The selection of the dimensions, 128 $\times$ 128, was based on a comparison with 256 $\times$ 256, which did not yield any noteworthy enhancements in model performance. Subsequently, the 2D slices for each CT were merged into a single 3D volume. To standardise the number of slices along the z-dimension of each 3D volume, various strategies were explored. Based on the results obtained from these investigations, we opted to apply a cubic interpolation method for all experiments. As a result, the final 3D volume comprises dimensions of 128 $\times$ 128 $\times$ 64. A summary of preprocessing is explained in Algorithm 1. Stratification was used to ensure each class exists in all three splits.

**Algorithm 1:**
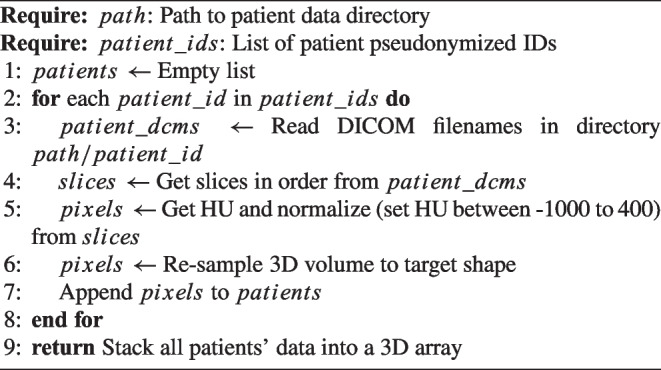
Read patient data and preprocess process

### Data Augmentation

Since we are working with a relatively small dataset, and DL models typically require a substantial amount of training data to effectively learn and train, we have employed data augmentation as a key data preprocessing technique in DL. This approach artificially enhances the diversity of the training dataset by applying various transformations to the input data. In our research, we have applied several augmentations to the training dataset to enhance the robustness and generalisation of our CNN models. To achieve this, we used TorchIO [24] to apply the following augmentations on-the-fly with PyTorch Dataloader using mini-batches of data:

1. RandomAffine transformation, which applies random geometric transformations to the 3D CT scans. This involves randomly rotating CT scan images between 1 and $10^{\circ }$ with a 20% probability of rotation.
2. RandomFlip transformation, which performs random flips along the first and second axes (axes 0 and 1) with a 40% probability of flipping.
3. RandomNoise transformation, introducing random noise to the CT scan data. The amount of added noise is controlled by the mean and standard deviation (std) parameters, with a mean of 0 and std of 0.05. This transformation has a 10% probability of adding random noise.
4. Z-score normalisation (Standardisation) was applied, where each 3D volume is scaled to have a mean of 0 and a standard deviation of 1. This normalisation aids in model convergence during training and improves training process stability. It was also applied to the validation and test sets.

**Fig. 4:**
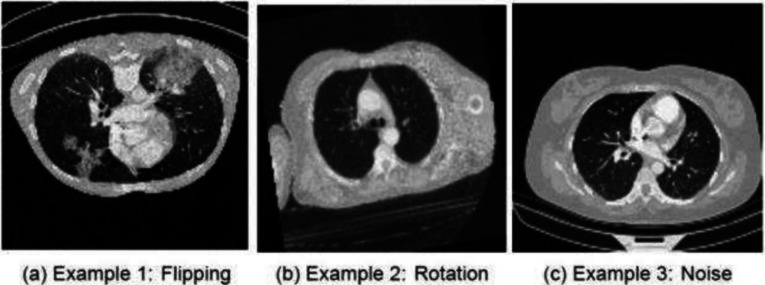
Augmented image examples

We provide an example of augmented slices from the training set in Fig. 4 below. As indicated in Table 2, the dataset exhibits a slight class imbalance issue. To address this, we employed a random data oversampling strategy to artificially increase the number of instances in the minority class. This oversampling was specifically applied to the training set, ensuring that the samples of all classes are equal to the class with the highest number of instances.

**Fig. 5:**
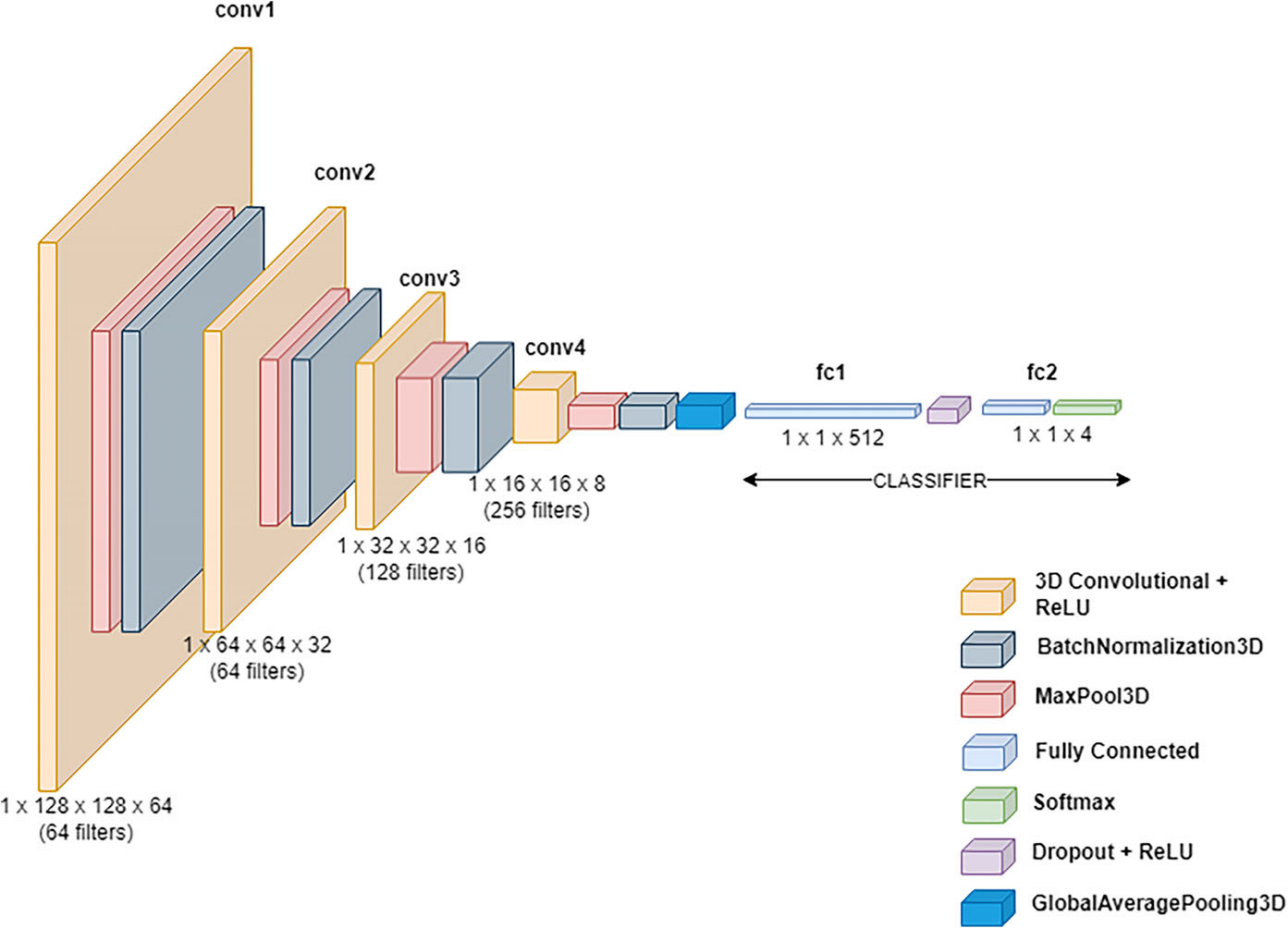
Architecture diagram for baseline 3D CNN

## Deep Learning Methods for BSTI COVID-19 Classification using CT Volumes

This study proposes a DL-based multi-class classification method that categorises CT volumetric images into four classes: ‘Classic’, ‘Probable’, ‘Indeterminate’, or ‘Non-COVID’ in alignment with BSTI. As far as we are aware, this study represents the first attempt to classify 3D CT images based on the BSTI guideline labels. Our investigation involved several 3D CNN-based models, including a baseline CNN model, 3D ResNet-18, 3D ResNet-34, and 3D ResNet-50. In the following subsections, we add the details of each of the developed models.

### Baseline 3D CNN

**The baseline 3D CNN model was our first choice for classifying the 3D CT images of COVID-19, as it has the ability to analyse volumetric data and capture spatial dependencies across slices. Unlike 2D CNN models, 3D CNN models can analyse the entire scan in a single pass, preserving contextual information essential for accurate diagnosis. Additionally, using a 3D CNN as a baseline provides a straightforward yet robust foundation for benchmarking and comparison against more complex deep learning models, ensuring a clear assessment of performance improvements in subsequent research.** For this, a baseline 3D CNN model has been designed and trained from scratch. The architecture diagram is illustrated in Fig. 5.

**Fig. 6:**
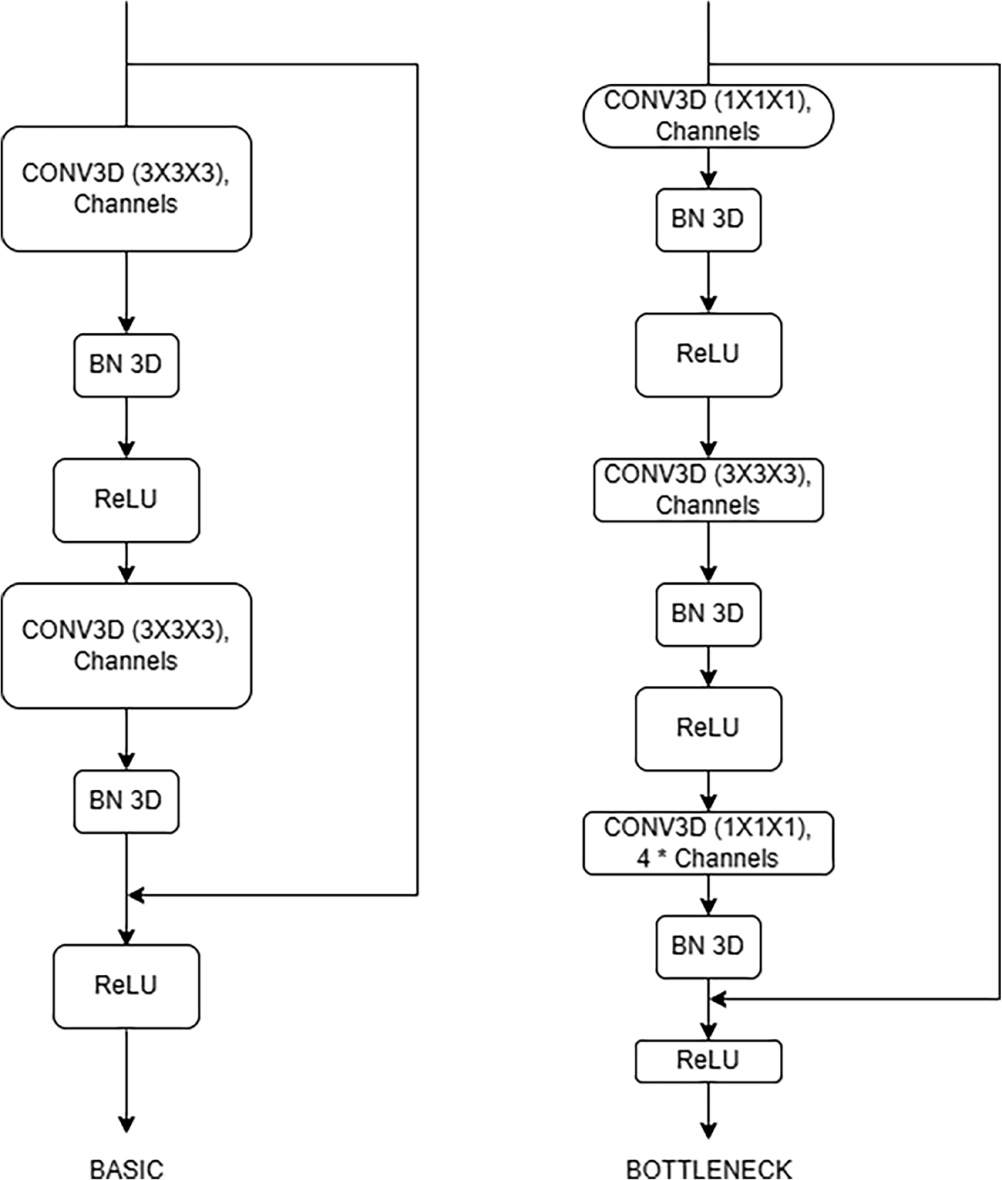
Basic block and bottleneck block for 3D ResNet-50 architecture. Kernel Size has been mentioned for each CONV3D and channels represent the feature channels

There are four convolutional blocks with each consisting of 3D convolution layer, Rectified Linear Activation Function(ReLU), 3D max-pooling layer, 3D batch normalisation layer. A kernel size of 3 and padding of 1 (to preserve spatial resolution) have been used for convolutional layers. ReLU was used as a non-linear activation function to activate all the convolutional layers. ReLU was chosen because it effectively addresses and mitigates the issue of vanishing gradients. Neural network models utilising ReLU tend to be more trainable and exhibit superior performance compared to models using alternative activation functions. Batch Normalisation reduces the internal covariate shifting which accelerates the model’s training. A 3D global average pooling is used at the end of the convolution blocks to convert each feature map into a single value. The classification layer at the end consists of two fully connected with dropout and ReLU in-between. Dropout helps in preventing overfitting and improving the performance of the model. The first fully connected consists of 512 layers and the second has layers equal to classification output classes (4 for multi-class) followed by the softmax function. The softmax layer converts the raw output or logits, from the final fully connected layer into a probability distribution over multiple classes. The overall number of training parameters is 1,353,412.

### 3D ResNet Architecture

**To improve the classification results obtained from the basedline 3D CNN model, we experimented with multiple 3D CNN-based architectures, including 3D DenseNet, and 3D VGG models. Based on our initial experiments, we found that 3D Residual Network (ResNet) consistently outperformed other models in terms of accuracy and robustness for classifying COVID-19 from 3D CT images.** 3D (ResNet) architecture has been adapted from the original 2D ResNet architecture [14] for processing 3D data. Unlike in 2D ResNet model, which utilises 2D convolutional layers, 3D ResNets employ 3D convolutional layers, which take into account spatial information across three dimensions. The key idea behind ResNet architectures is that they use residual blocks, where the original input is combined with the output of the residual block. This allows for the model to focus on learning the residual (difference) between the input and output. **The residual blocks help to mitigate the vanishing gradient problem and facilitates training deeper networks, making it particularly suitable for capturing the complex spatial patterns present in 3D medical data. Given its superior performance, we selected 3D ResNet as the primary model for the remainder of our analysis to ensure the most reliable results.**

In comparison to the baseline CNN architecture, ResNet models are known for their depth, which requires a large number of training parameters and more resources to train. The architecture of a 3D ResNet can vary in terms of the number of layers, block configurations, and hyperparameters [11]. In our study, we investigated three variations: 3D ResNet-18, 3D ResNet-34, and 3D ResNet-50 which proved to be successful in similar tasks.

**Fig. 7:**
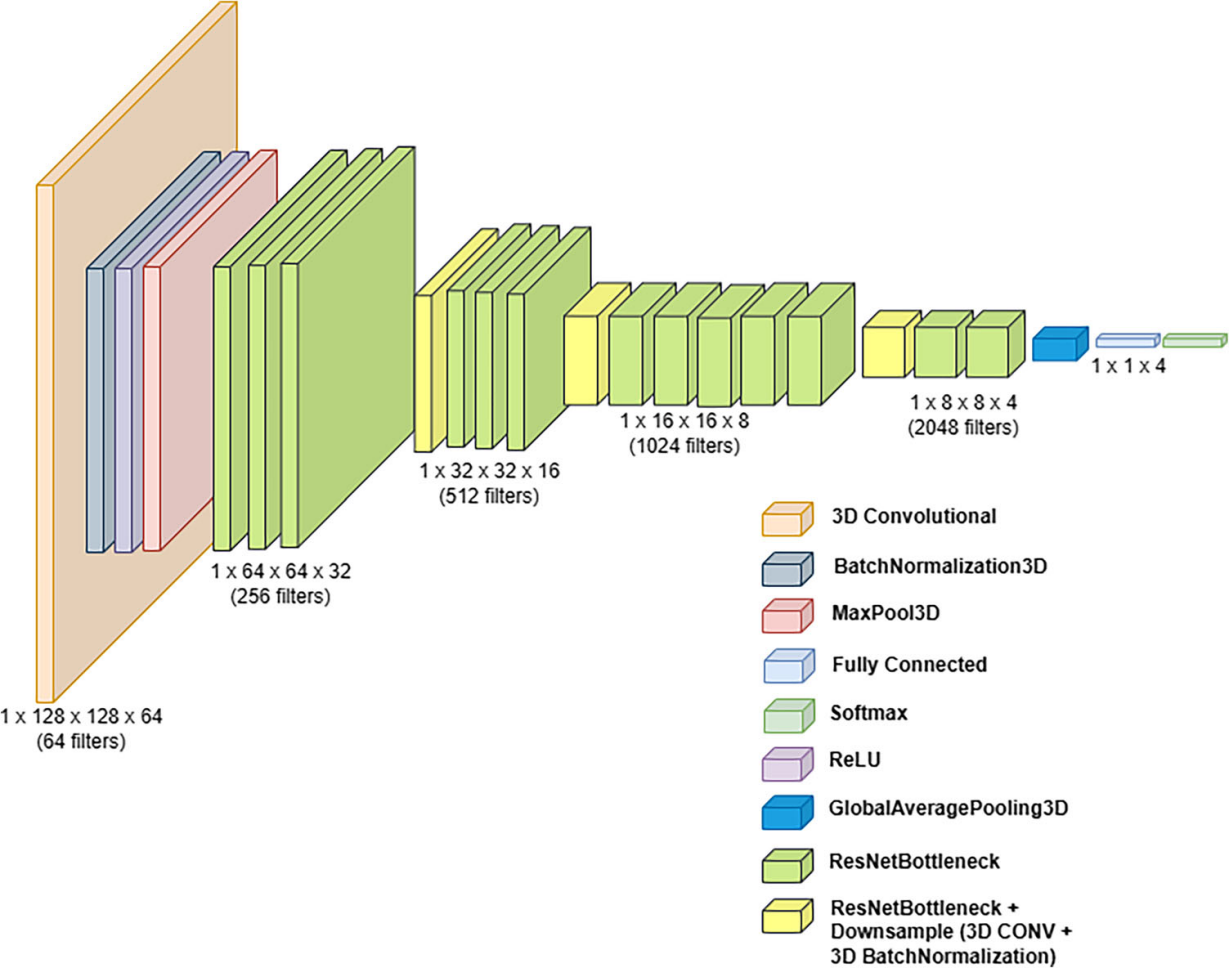
Architecture diagram for 3D ResNet-50

The 3D ResNet-18 and ResNet-34 make use of the Basic Block whereas 3D ResNet-50 uses Bottleneck Block as illustrated in Fig. 6 below. The connection shown in Fig. 6 shows a bypass that connects the top block to the layer before ReLU and helps in resolving the issue of vanishing gradient [14]. The basic and bottleneck blocks consist of 2 and 3 convolutional layers respectively with batch normalisation and ReLU in-between. Downsampling has been performed in the last convolutional layer of each basic or bottleneck block (except the first one) with a stride of 2. Monai [5] has been utilised to build the ResNet models.

The network architecture diagram for 3D ResNet-50 has been illustrated in Fig. 7. The ResNet-50 used the bottleneck block (see Fig. 6. Initially, a 3D convolutional layer is applied with single input channel, large kernel size of (7, 7, 7), and 64 output channels, followed by batch normalisation, ReLU activation, and Max pooling which reduces the spatial dimensions. This is followed by four layers, each containing multiple residual blocks (Bottleneck Blocks). Towards the end, AdaptiveAvgPool3d layer is used which further reduces the dimensions to a (1, 1, 1) size before reaching the final Linear layer (Classification Layer), which produces the output. The architecture for 3D ResNet-18 and 3D ResNet-34 are similar with fewer layers in comparison.

### Transfer Learning

Due to the limited amount of training data, transfer learning has been adopted to train the ResNet architectures to help improve the classification performance and lower the amount of training time. The ResNet models have been pre-trained on the Kinetics-700 dataset [6], which includes a collection of 650,000 RGB video clips covering 700 different human motion classes. Every clip has an action class annotation and is roughly ten seconds long. The pre-trained weights are used from the study in [18]. To pre-train the model, the original 3D ResNet architectures [11] was modified to replace the three channels (used for RGB video clips) in the first convolution layer with a single input channel as we are dealing with 16-bit grayscale images. The original weight for the three-channel layer was summed and used for the new single-channel convolution layer. The fully connected layer at the end was replaced to match the number of output classes in our dataset (4 or 3 in multi-class classification). In DL models for computer vision, initial layers are often used to learn image features, while the subsequent layers are related to instance categories. Deciding what layers to pre-trained in the DL network varies, depending on the data and task. In our case, the Kinetics-700 dataset is quite different to the 3D CT scan images (out of domain data). For this, we opted to train all layers in the ResNet models.

**Table 3:** Hyperparameter values used in the four-class classification problem, as per the BSTI

Early stopping	Batch	Optimizer	LR	Scheduler	Factor	Epochs
Patience 20 epochs	4	SGD	0.001	ReduceLR - OnPlateau	0.1 (Patience 10 epochs)	100

### Model Training Hyper-Parameters

Our tests were performed using Google Colab, (NVIDIA Tesla T4 GPU with 16 GB memory, Intel Xeon CPU with 2 vCPUs, and 13GB RAM Data). For each experiment, the data was stratified into 60% for training, 20% for test, and for 20% validation datasets respectively. For training and validating our models, an optimizer and suitable fit functions were used, where each model ran between 100 and 200 epochs with a batch size of 4 and 8. The optimizer used for our proposed models was the (Stochastic Gradient Descent (SGD)) as it proved to be effective for the given data and task. The optimal number of epochs has been decided based on monitoring each model’s performance on the validation set during training. The batch size represents the number of images used to train a single forward and backward pass. It has been observed during experiments that smaller batches have better generalizability optimization convergence. The learning rate controls how the model adjusts in response to low loss. Based on our empirical selection, a learning rate value of 0.001 was used with the SGD optimizer, allowing frequent reweighting of the model and low memory usage. To improve the training convergence and generalisation performance, ReduceLROnPlateau [25] from PyTorch was utilised. A value of 10 was used based on empirical selection. An early stopping criterion with the patience of 30 epochs (if no improvement in validation loss) was used to stop training and prevent overfitting. Additionally, a checkpoint to recover best-performing model weights is used. The criterion used is cross-entropy, which is a popular loss function that can be used for multi-class classification. An early stopping mechanism to prevent overfitting has been used, with the additional benefit of shorter training time required.

### Post-Hoc Visual Explainability

Gradient-weighted Class Activation Mapping (Grad-CAM) is an XAI algorithm used with CNN-based models to visualise the regions of an input image that contributed the most to the model’s decision [29]. This is done by calculating the gradient of the target class score with respect to the final convolutional layer of the neural network. This gradient information is then used to generate a heatmap, emphasising the regions that had the most influence on the model’s output.

## Experiments and Results

This section reports the evaluation of the 3D DL-based models (explained above) for the BSTI COVID-19 detection using CT volumes. The first experiment aims at classifying the images into **four** classes: ‘Classic’, ‘Probable’, ‘Indeterminate’, or ‘Non-COVID’, in line with BSTI. Based on the results obtained from this experiment, the best-performing model was retrained on three classes only: ‘Classic’, ‘Probable’, or ‘Non-COVID’. Finally, we apply visual explanations using Grad-CAM [29] to aid in understanding the models’ decisions. **All analyses were conducted at the case level, utilising the entire volumetric CT scans for both training and evaluation of the deep learning model**.

### Model Evaluation Metrics

**The performance of our models has been evaluated using several evaluation metrics, including (a) Accuracy, which is the ratio of correctly predicted observations to the total observations, (b) Precision, which is the ratio of correctly predicted positive observations to the total predicted positive observations, (c) Recall, which is the ratio of correctly predicted positive observations to all observations in the actual positive class, and (d) F1 score, which is the harmonic mean of Precision and Recall, providing a single score that balances both metrics. In addition, we evaluated the confusion matrix, which is a cross-table that records the number of occurrences between true/actual vs. predicted classification. Finally, we conducted statistical tests to compare the performance metrics (Precision, Recall, and F1 score) across the validation and test sets for each class. The null hypothesis** $\varvec{(H_0)}$ **assumes no significant difference between the two sets. A paired t-test** [16] **was applied for each metric and class, with a significance threshold of** ***p*** < **0.05.**

### Quaternary-Class Classification: Followingwing BSTI Reporting

Table 3 shows the optimal hyperparameters used for running the four-class classification experiments.

**Table 4:** Results obtained from the studied DL approaches (on the validation and test sets) for classifying the CT volumes into four BSTI COVID-19 groups

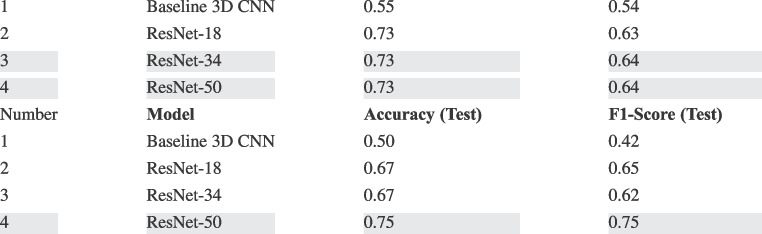

**Table 5:** Individual class classification performance of ResNet-50 on validation and test sets for categorising CT volumes into four COVID-19 BSTI groups

Class	Validation set			Test set		
	Precision	Recall	F1 score	Precision	Recall	F1 score
Non-COVID	0.50	0.67	0.57	0.75	1.00	0.86
Classic-COVID	1.00	1.00	1.00	1.00	1.00	1.00
Probable-COVID	1.00	1.00	1.00	1.00	0.60	0.75
Indeterminate	0.00	0.00	0.00	0.33	0.50	0.40
Accuracy	0.73			0.75		

The results, presented in Table 4, indicate that ResNet-50 outperforms the other three examined models across both the test and validation sets. Specifically, it attains an accuracy and F1 score of 73% and 64%, respectively, on the validation set, and 75% and 75%, respectively, on the test set. In contrast, the baseline 3D CNN falls behind, achieving an accuracy and F1 score of 55% and 54%, respectively, on the validation set and 50% and 42%, respectively, on the test set.

To gain a deeper understanding on the performance of the best-performing model (ResNet-50), we conducted an assessment of classification performance for individual classes, and results are reported in Table 5. Results show that the ResNet-50 model excels in identifying ‘Classic’ and ‘Probable’ COVID-19 groups. It exhibits moderate performance in detecting ‘Non-COVID’ cases. However, it encounters significant challenges in detecting the ‘Indeterminate’ COVID-19 cases.

**Fig. 8:**
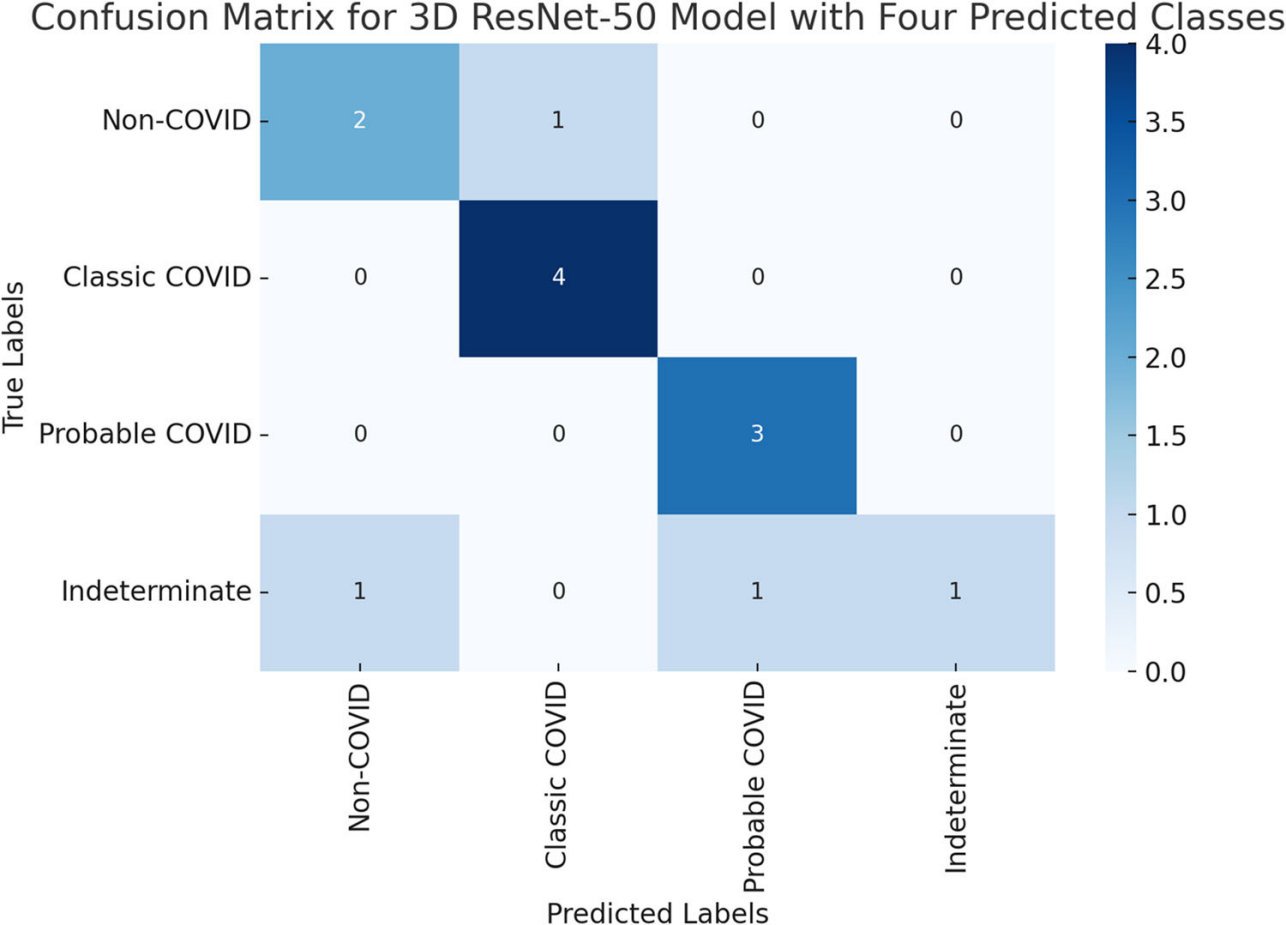
Confusion matrix results for quaternary-class classification obtained from the ResNet-50 (best-performing model)

**Figure** 8**displays the confusion matrix for the ResNet-50 model (best performing) on the test set, and it shows that ResNet-50 performs quite well in detecting ‘Classic-COVID’ and ‘Probable-COVID’. ‘Non-COVID’ cases are also reasonably detected. However, the model struggles with detecting the ‘Indeterminate’ class.**

Table 6 summarises the performance metrics and statistical significance of differences between the validation and test sets.

Key observations from the above table are:

- The Non-COVID class shows significant improvements in Precision, Recall, and F1 score from the validation to the test set.
- The Classic-COVID class maintains consistent performance across sets, with no significant differences observed.
- The Probable-COVID class shows a significant decrease in Recall and F1 score on the test set.
- The Indeterminate class shows significant improvements in all metrics, albeit from low values in the validation set.

### Ternary-Class Classification: Excluding the ‘Indeterminate’ COVID-19 Cases

The above results reveal that the four-class classification DL models struggled to detect the ‘Indeterminate’ class. In this experiment, we trained the best-performing DL architecture (ResNet-50) to classify each CT image into: ‘Classic’, ‘Probable’, or ‘Non-COVID’. To achieve this, we excluded the CT images associated with the ‘Indeterminate’ class from the original dataset, transforming the classification problem from a quaternary to a ternary class challenge.

The ResNet-50 model was trained using optimal hyperparameters outlined in Table 7. The classification performance of the model, as well as its performance on individual classes, are detailed in Table 8. When compared to the four-class classification model, results of the ternary-class model demonstrate significant improvement in performance after excluding the ‘Indeterminate’ class from the dataset, achieving an accuracy of 90% on the test set.

**Table 6:** Statistical comparison of metrics between validation and test sets for the quaternary-class classification

Class	Metric	*p*-value	Significant
Non-COVID	Precision	0.041	Yes
	Recall	0.032	Yes
	F1 score	0.029	Yes
Classic-COVID	Precision	–	No
	Recall	–	No
	F1 score	–	No
Probable-COVID	Precision	–	No
	Recall	0.018	Yes
	F1 score	0.022	Yes
Indeterminate	Precision	0.049	Yes
	Recall	0.045	Yes
	F1 score	0.047	Yes

*Yes—indicates statistically significant differences* (*p* < 0.05)

**Table 7:** Hyperparameter values used in the ternary-class classification problem (using ResNet-50 model) after excluding the ‘Indeterminate’ class

Early stopping	Batch	Optimizer	LR	Scheduler	Factor	Epochs
Patience 30 epochs	4	SGD	0.001	ReduceLR - OnPlateau	0.1 (Patience 10 epochs)	100

**Table 8:** Individual class classification performance of ResNet-50 on validation and test sets for categorising CT volumes into three COVID-19 groups, excluding the ‘Indeterminate’ class

Class	Validation set			Test set		
	Precision	Recall	F1 score	Precision	Recall	F1 score
Non-COVID	1.00	1.00	1.00	0.75	1.00	0.86
Classic-COVID	1.00	0.50	0.67	1.00	1.00	1.00
Probable-COVID	0.80	1.00	0.89	1.00	0.80	0.89
Accuracy	0.89			0.90		

**Fig. 9:**
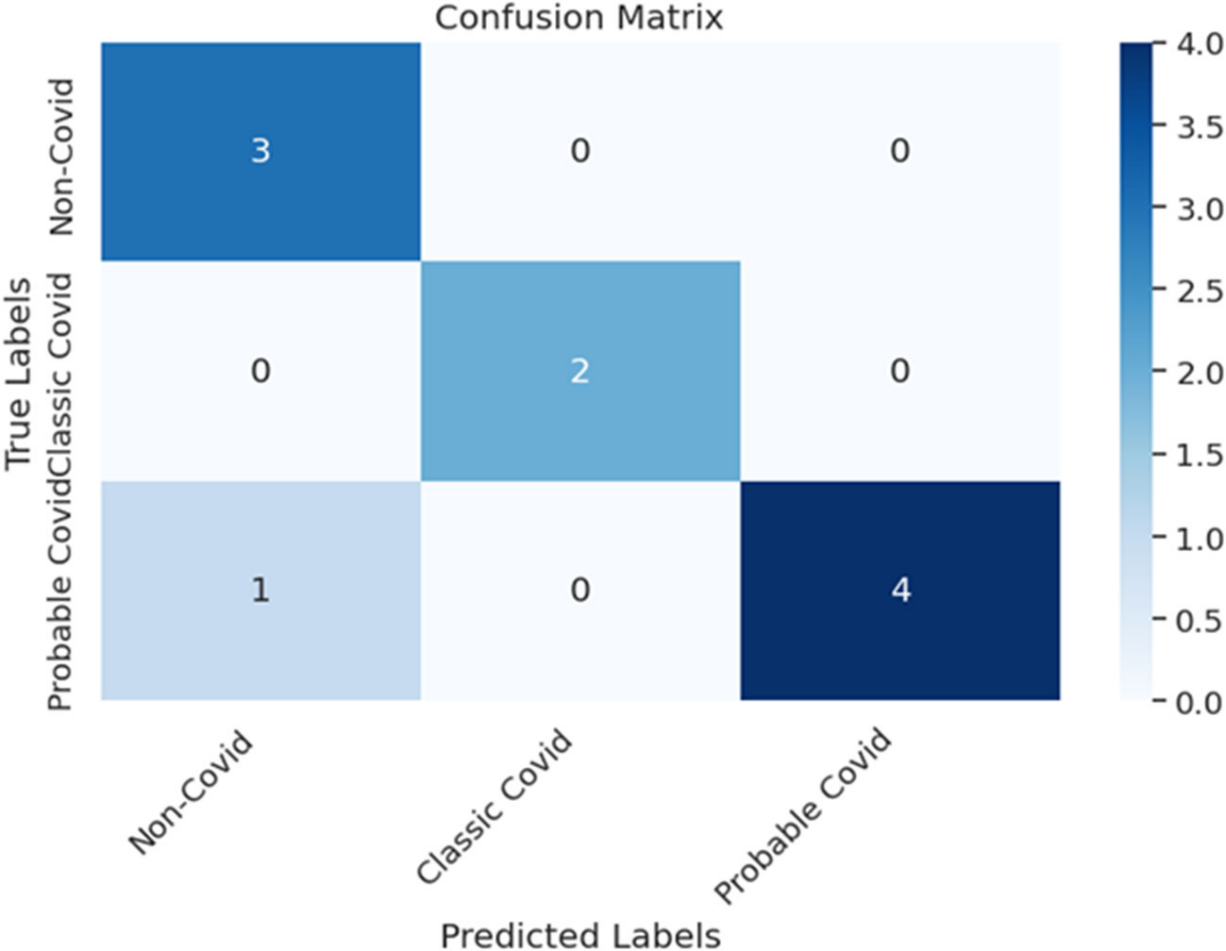
Confusion matrix results for ternary-class classification obtained from the ResNet-50 (best-performing model)

**Figure** 9**displays the confusion matrix for the ResNet-50 model (best performing) on the test set, and it shows that ResNet-50 performs quite well in detecting classic COVID and Non-COVID classes. In addition, probable cases (majority class) are reasonably detected.**

Table 9 summarises the performance metrics and statistical significance of differences between the validation and test sets.

Key observations from the above table are:

- For the Non-COVID class, significant differences were observed in Precision and F1 score.
- For the Classic-COVID class, significant differences were noted in Recall and F1 score.
- For the Probable-COVID class, significant differences were observed in Precision and Recall.

### Post-Hoc Visual Explanation Results

After obtaining the classification results and to further improve the interpretability of the models, we applied two XAI methods, Grad-CAM (explained above), to the best-performing model (ternary-class classification ResNet-50 model). The purpose of this step is to enhance the transparency and trustworthiness of the 3D classification model, which could help clinicians to verify and validate the model’s decisions, ultimately improving trust and deployment of DL-based techniques in clinical settings [3, 9, 10].

**Fig. 10:**
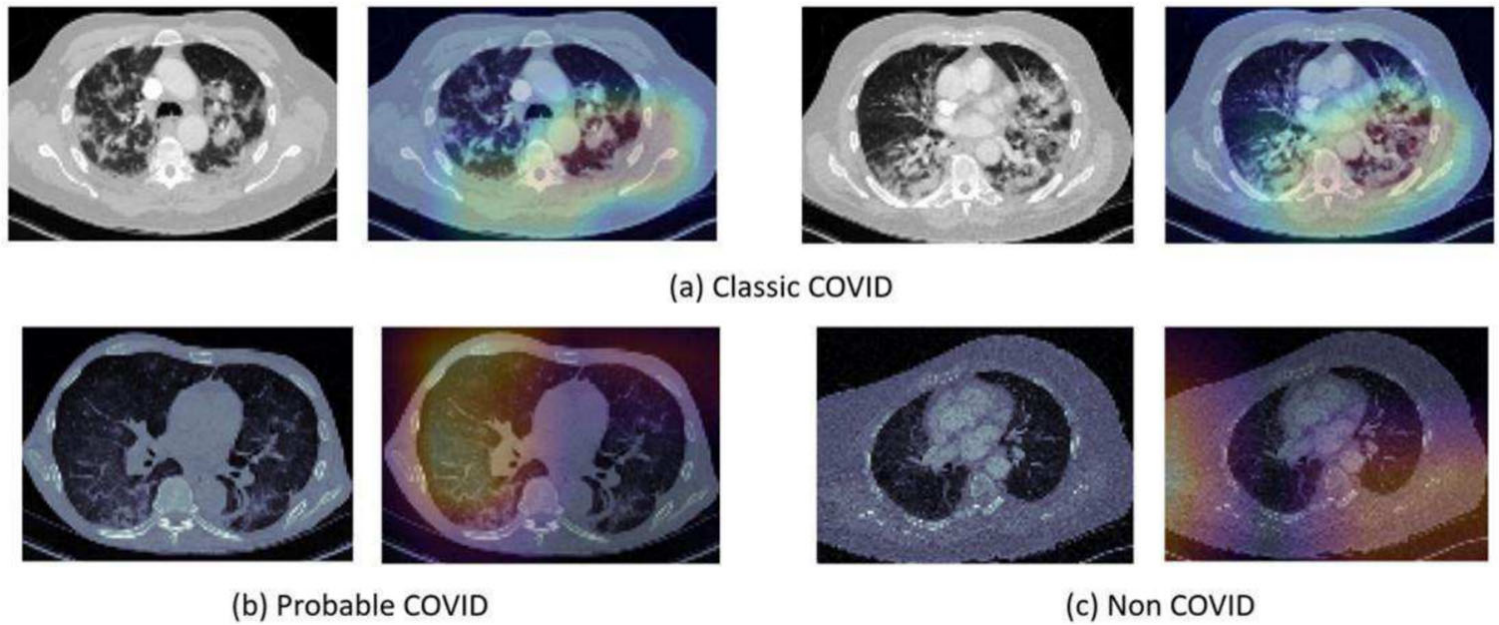
Post-hoc visual explanation results of the Grad-CAM explanation technique (using ResNet-50 model) for four examples CT test slices correctly predicted as **a** ‘Classic-COVID’, **b** ‘Probable-COVID’, and **c** ‘Non-COVID’. Image pairs show original preprocessed images on the right and Grad-CAM explanation (heatmap highlighting the important areas in the image) on the left

Figure 10 shows Post-hoc visual explanation results for three example CT test slice predicted as (a) ‘Classic-COVID’, (b) ‘Probable-COVID’, and (c) ‘Non-COVID’. To generate the Grad-CAM results, we obtained the activation maps generated by the last layer of the ResNet-50 model using the Grad-CAM algorithm and the Captum’s library. **Unlike in Fig.** 10**c ‘Non-COVID’, in Fig.** 10**a and b, the Grad-CAM heatmap generated from the CT image highlights key regions associated with COVID-19, such as ground-glass opacities or other areas of lung consolidation commonly recognised. All these biologically relevant areas relate to the inflammation and fluid buildups in lung tissues caused by the viral infection itself in line with clinical observation. However, it misses certain affected areas in Fig.** 10**a, which might be due to inherent limitations of model sensitivity and the spatial resolution of activation maps. This partial detection then shows that model-generated output should be complemented and reviewed by clinical expertise if a diagnosis is to be made and also indicates that further refinement of interpretability methods is needed to capture all relevant pathological features from imaging data** [9, 10].

## Discussion

In this study, we collected a unique dataset of experts labelled 56 CT volumes from patients suspected to have COVID-19 infections. Images were annotated by expert radiologists into four classes, following the BSTI. Numerous studies have been proposed in the literature for detecting COVID-19 using binary classification (positive or negative) [32, 36] or multi-class classification solutions [7, 15, 17, 22, 26, 28, 30, 31, 34]. However, to the best of our knowledge, classifying CT volumes using the BSTI COVID-19 reporting system has not been presented in the literature. Our target was to develop a DL-enabled diagnostic system that can automatically detect the BSTI group from CT volumes. To this end, we studied the performance of four 3D DL architectures, including ResNet models pre-trained on the Kinetics-700 video dataset. In advance of the machine learning analysis, we applied best practices in data pre-processing, data augmentation, and model optimisation to avoid overfitting issues, enhance accuracy, and improve model generalisation.

The 3D DL models were compared in terms of accuracy, recall, precision, and F1 score. In the context of four-class classification, our results show that the ResNet-50 model gave the best overall classification performance, achieving an accuracy of 75% on the test data set. While the baseline 3D CNN was the least performing, achieving an accuracy of 50% on the test data set. This is because, unlike the deep architecture used in ResNet-based models, the CNN model included a smaller number of layers, restricting the ability to capture feature details in the image that deeper models can do.

Inspecting the classification performance of individual classes revealed that ResNet-50 (best-performing model) was able to accurately detect the ‘Classic’ and “Probable” COVID-19 groups, with a recall of 100% on the validation set. This maybe due to the presence of COVID-19 markers, such as GGOs, in the CT images of those two BSTI COVID-19 categories. The ability to automatically detect ‘Classic’ and ‘Probable’ COVID-19 cases correctly is crucial, as it allows instant interventions, especially for critical cases. On the other hand, the ResNet-50 model completely failed to detect the ‘Indeterminate’ COVID-19 group, which was often misclassified as ‘Non-COVID’. To investigate the impact of the underperforming class (in this case, ‘Indeterminate’) on the model performance, we removed the CT images associated with the ‘Indeterminate’ group and retrained the ResNet-50 model to classify the data into three classes. The ternary classification model yielded an average accuracy of 90%, which was significantly higher than the average accuracy obtained from the quaternary classification model of 75%. In the context of machine learning, removing the underperforming class from the dataset can lead to better decision boundaries and improved classification accuracy.

This study employs a novel dataset based on BSTI, therefore, direct comparison with current methods was infeasible. Table 10 provides a comparison with other multi-class classification methodologies for COVID-19 detection, which reveals that our results are comparable to other studies for COVID-19 severity classification. However, as indicated previously, our study proposes a unique contribution by considering the labelling of the BSTI system.

**Table 9:** Statistical comparison of metrics between validation and test sets for the ternary-class classification

Class	Metric	*p*-value	Significant
Non-COVID	Precision	0.032	Yes
	Recall	1.000	No
	F1 score	0.045	Yes
Classic-COVID	Precision	1.000	No
	Recall	0.023	Yes
	F1 score	0.018	Yes
Probable-COVID	Precision	0.041	Yes
	Recall	0.039	Yes
	F1 score	1.000	No

*Yes—indicates statistically significant differences* (*p* < 0.05)

**Table 10:** Comparison with other methodologies for multi-class classification

Modality	Model	Category	Results	Paper
X-ray	DenseNet-121 & Multi-task learning	Negative, Typical, Indeterminate, or Atypical appearance of COVID-19 [21]	Accuracy: 64% F1 score: 46%	Muhammad et al. [26]
X-ray	Lightweight ResGRU	Negative, Typical, Indeterminate, or Atypical appearance of COVID-19 [21]	Accuracy: 80.7% F1 score: 80.5%	Ahmad et al. [1]
X-ray	EfficientNetv2-L	Negative, Typical, Indeterminate, or Atypical appearance of COVID-19 [21]	Accuracy: 70% F1 score: 80.5%	Khan et al. [19]
CT	Multinomial Logistic Regression (MLR)	Severe, Moderate, Mild, and Normal [22]	Accuracy: 92%	Shiri et al. [30]
CT	Pretrained 3D ResNet-34	Classic, Probable, Indeterminate, and Non-COVID [8]	**Accuracy: 75% F1 score: 75%**	**Proposed Study**
CT	Pretrained 3D ResNet-50	Classic, Probable, and Non-COVID	**Accuracy: 90% F1 score: 92%**	**Proposed Study**

To improve the explainability of our framework, we have applied Grad-CAM, which provided visual explainability outcomes for positive COVID-19 cases. Our results reveal that Grad-CAM was able to visualise regions of the infected areas (inline with the ‘Classic-COVID’ markers (see Table 2).

**While several studies aim to automate severity scale detection for COVID-19, a key contribution of this paper is the identification of different COVID-19 types using the BSTI COVID-19 reporting system — a standardised framework widely adopted in the UK for categorising imaging findings. This system classifies chest X-ray and CT findings into four main categories, ensuring consistency and comparability across healthcare institutions. By leveraging this approach, our work enhances the standardisation of COVID-19 imaging reports, improving diagnostic efficiency and offering a robust foundation for future responses to respiratory pandemics.**

## Limitations and Future Work

**The main limitation of this study is the poor automatic detection of the ‘Indeterminate’ COVID-19 cases in volumetric CT images. This is mainly due to the inherent challenges associated with the ‘Indeterminate’ COVID-19 cases as they represent a gray area where definitive diagnosis is difficult, even for experienced radiologists. Indeterminate cases often share characteristics with the three other classes of ‘Classic-COVID’, ‘Probable-COVID’, and ‘NON-COVID’, making automatic detection challenging. The other contributing factor would be the limitation in training data size which may hinder the algorithm’s ability to learn and accurately identify these cases. Additionally, the complexity of volumetric CT images adds another layer of complexity compared to 2D chest X-rays, potentially making it more difficult for automated systems to capture the nuanced features of ‘Indeterminate’ cases. To address this limitation, our future research could focus on developing more advanced deep learning models, incorporating larger training data, and refining the definition and characteristics of indeterminate cases to improve automatic detection accuracy.**

Interestingly, the poor detection accuracy of ‘Indeterminate’ COVID-19 cases is consistent with recent research in [23], which aimed to assess readers agreements on the BSTI diagnostic classification for COVID-19 based on chest radiographs (CXR). Twenty readers, including consultant chest radiologists, general consultants, specialist registrar radiologists, and infectious diseases clinicians, evaluated 305 CXRs from 176 COVID-19 patients. The agreement for categorising indeterminate CXRs requiring CT imaging was low (28–37%), and the highest agreement was observed for classic/probable categories (66–76%). These findings were also consistent with the research in [13], which aimed at validating the BSTI reporting for categorising chest radiographs in COVID-19 reporting, assessing reproducibility amongst radiologists, and diagnostic performance. Seven consultant radiologists evaluated chest radiographs from 50 COVID-19 patients and 50 control patients with COVID-19-like symptoms. The results demonstrated excellent specificity (100%) and moderate sensitivity (44%) for Classic/Probable-COVID. Yet, fair agreement was observed for ‘Indeterminate for COVID-19’ (*k* = 0.23) and ‘Non-COVID-19’ (*k* = 0.37) categories.

**This study employed data augmentation techniques, including flipping, rotation, and intensity adjustments, to address the limited size of the training set. While these methods are standard in medical image analysis, they inherently produce augmented images that are correlated with the original data. As a result, there is a potential risk of the model learning features specific to the augmented variations rather than generalising to independent cases. This limitation may contribute to an overestimation of model performance. Future work should focus on expanding the dataset to include more diverse, independent samples and exploring advanced augmentation techniques, such as generative adversarial networks (GANs), to create synthetic but uncorrelated training data,**

Our study encountered another limitation, which is the exclusive reliance on single-centre data. However, it is noteworthy that the training dataset was thoughtfully curated to encompass a diverse representation of ethnicities in the West Midlands region. Future research endeavours will address these issues by incorporating large-scale, multi-centric datasets and images from diverse observers to enhance model training and broaden generalizability. Additionally, we aim to assess alternative deep learning architectures, especially in the context of multi-classification tasks involving 3D images.

## Conclusion

In this study, a novel multi-classification DL model was designed and evaluated for detecting COVID-19 categories using BSTI reporting guidance for radiologists for CT studies. While numerous studies propose COVID-19 detection through binary or multi-class classification, there is a lack of literature on classifying CT volumes using this BSTI reporting guidance.

Four DL architectures were presented and evaluated, including DL ResNet pre-trained models. Our experiments and results performed revealed that ResNet-50 model outperformed the other three experimented models and it achieved 75% accuracy in four-class classification task. To assess the impact of the ‘Indeterminate’ class on model performance, we excluded CT images associated with this class and retrained the ResNet-50 model for three-class classification, resulting in a significantly higher accuracy of 90% compared to the four-class model. Our models show excellent automatic detection for ‘Classic’ and ‘Probable’ BSTI COVID-19 categories (with 100%) with poor detection ability for ‘Indeterminate’ COVID-19 cases. These findings are consistent with other clinical researchers which aimed at validating the BSTI reporting manually amongst consultant radiologists.

Finally, we investigated the use of the Grad-CAM method to improve the interpretation of the proposed DL model. This is done by highlighting (via heatmap) the critical parts of the image that make the DL decision, and therefore providing useful insights to the medical staff. Visual aids can help clinicians identify and evaluate key symptoms of COVID-19 (such as GGOs) on CT images.

## Data Availability

The data related to this manuscript cannot be publicly available to protect patients’ data, in compliance with the Sandwell and West Birmingham Hospitals NHS Trust data processing agreement statement. The Python code is available at this link

## Footnotes

1 Available from: https://mircwiki.rsna.org/index.php?title=MIRC_CTP
